# Epigenetic reprogramming of the zygote in mice and men: on your marks, get set, go!

**DOI:** 10.1530/REP-16-0376

**Published:** 2016-10-20

**Authors:** Rupsha Fraser, Chih-Jen Lin

**Affiliations:** The University of EdinburghMRC Centre for Reproductive Health, Queen’s Medical Research Institute, 47 Little France Crescent, Edinburgh, EH16 4TJ, Scotland, UK

## Abstract

Gametogenesis (spermatogenesis and oogenesis) is accompanied by the acquisition of gender-specific epigenetic marks, such as DNA methylation, histone modifications and regulation by small RNAs, to form highly differentiated, but transcriptionally silent cell-types in preparation for fertilisation. Upon fertilisation, extensive global epigenetic reprogramming takes place to remove the previously acquired epigenetic marks and produce totipotent zygotic states. It is the aim of this review to delineate the cellular and molecular events involved in maternal, paternal and zygotic epigenetic reprogramming from the time of gametogenesis, through fertilisation, to the initiation of zygotic genome activation for preimplantation embryonic development. Recent studies have begun to uncover the indispensable functions of epigenetic players during gametogenesis, fertilisation and preimplantation embryo development, and a more comprehensive understanding of these early events will be informative for increasing pregnancy success rates, adding particular value to assisted fertility programmes.

## Introduction

**Figure 1:**
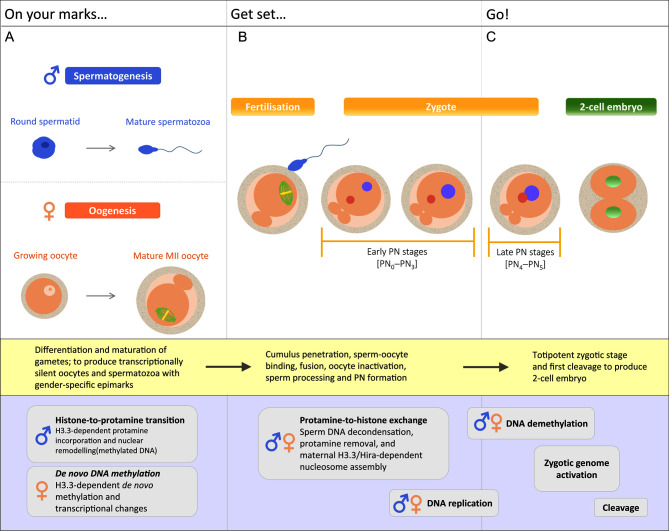
Overview of the cellular [yellow box ] and molecular [lilac box ] events during (A) gametogenesis (differentiation and maturation), with histone-to-protamine transition and nuclear remodelling in the paternal genome, and *de novo* methylation and transcriptional changes in the maternal genome; (B) fertilisation, with protamine-to-histone exchange, nucleosome assembly and PN formation, and DNA replication; and (C) preimplantation embryonic development, with DNA demethylation, two waves of zygotic genome activation to give rise to the transcriptionally active totipotent zygotic state and first cleavage to produce a two-cell embryo. ♂ paternal genome; ♀ maternal genome.

Life begins at fertilisation, the process when two gametes (sperm and oocyte) unite. A successful fertilisation event and subsequent embryonic development are dependent on the acquisition of developmental competence via highly orchestrated cellular and molecular events during gametogenesis. Before fertilisation, sperm and oocyte genomes are transcriptionally silent as a consequence of hypermethylation of their respective genomes, which ensures the repression of pluripotent markers (Seisenberger *et al*. 2013) (Fig. 1a). Upon fertilisation, extensive epigenetic reprogramming takes place, whereby the two highly differentiated gametes come together and reorganise their cellular and molecular signatures by global DNA demethylation (Fig. 1b) to establish a transcriptionally activated, totipotent zygote (Biechele *et al*. 2015) (Fig. 1c).

Epigenetic regulation describes the hereditary genetic changes that are caused by mechanisms other than modifications in underlying DNA sequences, and epigenetic regulators can influence both transcriptional and post-transcriptional gene expression. Nucleosomes are octamers formed by two molecules of each of the canonical core histones H2A, H2B, H3 and H4, whereas the linker histone H1 binds to the nucleosomal and linker DNA (Talbert & Henikoff 2010, Kowalski & Palyga 2012, Rathke *et al*. 2014). Histones can yield variations in the chromatin structure by the incorporation of histone variants, producing dynamic patterns of transcriptional regulation. Histone variants refer to non-canonical (non-allelic) variants of the core histones with very small minor amino acid variations, which can cause dynamic changes in protein expression, regulation and function from canonical counterparts. For example, there is only a difference of 4−5 amino acids between the core histone H3 and its variant H3.3 (Maze *et al*. 2014). Their functions will be discussed in detail in this review.

DNA methylation (5-cytosine methylation), by the addition of a methyl group to form 5-methylcytosine (5mC), is an epigenetic mark predominantly located at cytosine-phosphate-guanine (CpG) dinucleotides and is typically associated with gene silencing (Saitou *et al*. 2012, Ross & Canovas 2015, Schultz *et al*. 2016). The establishment and maintenance of DNA methylation patterns are implemented by DNA methyltransferases (Dnmt) 1, 3a and 3b (Seisenberger *et al*. 2013, Biechele *et al*. 2015). Demethylation describes the sequential oxidation of 5mC to 5-hydromethylcytosine (5hmC), 5-formylcytosine (5fC) and 5-carboxymethylcytosine (5caC) by the ten-eleven translocation (Tet) family of dioxygenases, Tet1−3 (Ito *et al*. 2010, Gu *et al*. 2011, Canovas & Ross 2016). Moreover, the involvement of thymine DNA glycosylase (TDG) and base excision repair (BER) activity has also been implicated in the highly coordinated process of post-fertilisation DNA demethylation (Guo *et al*. 2014, Lim *et al*. 2016, Weber *et al*. 2016). In addition, small RNAs, which are short non-coding RNA molecules typically 18−32 nucleotides in length, can regulate DNA methylation (Kuramochi-Miyagawa *et al*. 2008, Thomson & Lin 2009), suppress/destabilise mRNA (Djuranovic *et al*. 2012, Novina & Sharp 2004), and have been demonstrated as robust regulators of epigenetic reprogramming events (Yao *et al*. 2015, Dallaire & Simard 2016). There are three major classes of regulatory small RNAs, including microRNAs (miRNAs), endogenous small interfering RNAs (endo-siRNAs) and piwi-interacting RNAs (piRNAs) (Cook & Blelloch 2013). Here, we will focus on the most pertinent small RNAs and their functional roles in fertilisation and preimplantation development in mice.

In this review, we define the epigenetic reprogramming events that take place during gametogenesis, fertilisation and preimplantation development, in the following three stages, with particular emphasis on the dynamics of DNA demethylation and the role of histone variant H3.3: (1) On your marks, (2) Get set, (3) Go. The first stage explains the establishment of gamete-specific epigenomes during spermatogenesis and oogenesis. The next stage describes fertilisation and the subsequent chromatin remodelling in the early zygote. The third and final stage describes two critical reprogramming events, DNA demethylation and zygotic genome activation, which ultimately give rise to the establishment of a pluripotent embryo.

## On your marks…

### Preparation of parental genomes – spermatogenesis

The germ cell developmental process is controlled by a combination of genetic and epigenetic mechanisms. Spermatozoa are produced from male primordial germ cells (PGCs) that arise from progenitor cells during early embryonic development (Saitou *et al*. 2012, Bao & Bedford 2016). Global demethylation of previously acquired methylation patterns during early development takes place in PGCs, which then transition into spermatogonial stem cells (SSCs) that establish spermatogenesis (Manku & Culty 2015, Bao & Bedford 2016). Spermatogenesis takes place in three phases that include self-renewal of spermatogonia through mitosis, followed by meiosis of spermatocytes to form haploid spermatids and transformation of spermatids into spermatozoa by means of spermiogenesis (Yao *et al*. 2015). In humans, spermatogenesis can take 42−76 days to complete, with considerable variation between individuals (Misell *et al*. 2006), whereas in mice, it takes approximately 34.5 days (Oakberg 1956). Firstly, during the spermatogonial phase, SSCs residing on the seminiferous tubule basement membrane, divide by mitosis to form spermatogonia (Manku & Culty 2015). Next, diploid cells created during the spermatogonial phase give rise to haploid round spermatids by means of two sets of meiotic divisions (Yao *et al*. 2015). The spermatogonia incorporate testis-specific histone variants into their chromatin, and synthesis and deposition of these histone variants peak during this stage (Johnson *et al*. 2011, Rathke *et al*. 2014, Weber *et al*. 2016). During sperm head formation, compaction of the chromatin takes place as nuclear proteins are altered to increase the state of nuclear condensation (Hecht 1998, Wu & Chu 2008). Finally, there is spermiogenesis that involves the maturation and differentiation of the spherical, haploid spermatids into elongated, flagellated sperm. As they leave the testes and pass through the epididymal segments, spermatozoa are subjected to functional and morphological changes. Fully mature spermatozoa are stored in the tail of the epididymis, until they are ejaculated from the vas deferens (Rathke *et al*. 2014, Lehti & Sironen 2016).

It has been proposed that the final maturation from round spermatid to mature spermatozoa is only required in the transportation purposes to reach the oocyte (Practice Committee of American Society for Reproductive & Practice Committee of Society for Assisted Reproductive 2008). Round spermatids have a haploid genome that has completed paternal imprinting in mice, suggesting that the spermatid genome is genetically and epigenetically competent for embryonic development to term (Kimura & Yanagimachi 1995, Shamanski *et al*. 1999), but the exact stage of genomic imprinting in human spermatogenesis is unclear. In recent years, round spermatid injection (ROSI) has been developed as one of the routes of assisted fertilisation in cases of spermatogenic failure in both human and animal assisted reproductive technologies (ART), albeit with limited success (Gianaroli *et al*. 1999, Schoysman *et al*. 1999, Tesarik *et al*. 2000). The developmental efficiency of round spermatids is suboptimal compared with that of intracytoplasmic sperm injection (ICSI)-derived embryos across various species (Kimura & Yanagimachi 1995, Practice Committee of American Society for Reproductive & Practice Committee of Society for Assisted Reproductive 2008). Furthermore, a recent study demonstrated that ROSI-generated embryos can fail to undergo asymmetric active DNA methylation, although a causal association between impaired active DNA demethylation and reduced developmental aptitude observed in ROSI-derived embryos remains to be determined (Kurotaki *et al*. 2015), and ROSI-generated early embryos show aberrant gene expression patterns and increased aneuploidy incidence (Hayashi *et al*. 2003, Yamagata *et al*. 2009).

Spermatogenesis is characterised by histone-to-protamine transition (Fig. 1a). Ordered histone replacement and extensive nuclear remodelling take place, whereby histones are initially replaced with transition nuclear proteins (TNPs) and subsequently by protamines (small arginine-rich nuclear proteins that allows strong DNA binding), and the genome is packaged into protamine-associated, highly stable and compacted DNA (Carrell *et al*. 2007, Rathke *et al*. 2014). Once the protamines are incorporated, the paternal genome is further stabilised by the formation of disulphide bridges, thus further compressing the genome (Balhorn 2007). The transcriptionally quiescent genome now provides a hydrodynamic structure due to the reduction in nuclear shape and size, with potential for movement (Johnson *et al*. 2011). This exceptionally well-packaged design of the sperm nucleus (which is now 1/13th the size of an oocyte nucleus) confers protection of the paternal genome, making it resilient while passing through the female reproductive tract and resistant to nuclease attack, irradiation and shearing forces (Kuretake *et al*. 1996, Wykes & Krawetz 2003, Balhorn 2007, Miller *et al*. 2010, Johnson *et al*. 2011). In addition to serving protective purposes, it has been suggested that protamines may also be involved in epigenetic regulation and early embryogenesis (Balhorn 2007). However, 10−15% of the human sperm genome (1% in mice) retains a histone-bound nucleosomal structure (Brykczynska *et al*. 2010, Johnson *et al*. 2011), and histones carry out post-translational modifications that are transmitted to the early zygote and persist in the early embryo (van der Heijden *et al*. 2008, Vavouri & Lehner 2012, Rathke *et al*. 2014).

Histone variants play a key role in protamine transition and chromatin reorganisation during spermatogenesis, and variants of histones H1, H2A, H2B and H3 expressed in male germ cells may support the preparation of the chromatin structure for histone-to-protamine transition (Rathke *et al*. 2014). Some H1 variants (H1t, H1T2 and H1LS1) are testis-specific in mammals (Bao & Bedford 2016). H1t is expressed in spermatocytes and is present until post-meiotic chromatin reorganisation. H1T2 is expressed in male germ cells until the time of histone-to-protamine transition, thereby indicating its role in the replacement of histones by protamines, and H1T2 loss induces post-meiotic nuclear condensation defects and promotes reduction in fertility (Martianov *et al*. 2005, Tanaka *et al*. 2005, Bao & Bedford 2016). H1LS1 is highly expressed in spermatid nuclei and may be involved in late-stage spermiogenesis and histone replacement (Bao & Bedford 2016). Testis-specific variants of H2A and H2B (TH2A and TH2B) have also been detected in post-meiotic spermatids (Shinagawa *et al*. 2015), and expression of the spermatid-specific H2B (ssH2B) variant begins to decline before chromatin compaction and may be involved in transcriptional regulation (Chadwick & Willard 2001, Eirin-Lopez *et al*. 2008, Rathke *et al*. 2014). In addition, H2A.B.bd is strongly expressed in both mouse and human testes, has been observed in nucleosomal chromatin fraction of human sperm and may aid in chromatin reorganisation and histone displacement by TNPs (Ishibashi *et al*. 2010, Rathke *et al*. 2014, Bao & Bedford 2016). Perhaps the most important histone in epigenetics is H3. A number of H3 histone variants, including H3.1, H3.2, H3.3, H3t and H3.5, have been detected in mammals (Rathke *et al*. 2014), and H3.5 has been identified in human seminiferous tubules (Schenk *et al*. 2011). H3t is enriched in male germ cells, with synthesis taking place in spermatogonia, and it persists in detectable levels in spermatocytes and early spermatids (Trostle-Weige *et al*. 1984). H3.3 plays vital roles in regulating genome function and stability and is encoded by two conventional intron-containing genes *H3f3a* (*H3.3A*) and *H3f3b* (*H3.3B*). Messenger RNA (mRNA) expression of the former is found in pre-meiotic male germ cells and of the latter in meiotic prophase germ cells (Bramlage *et al*. 1997). A recent study in mice investigating the effects of null mutations in each of these genes has demonstrated that H3.3 is crucial for spermatogenesis, as *H3.3A*-mutant males were subfertile, with dysmorphic spermatozoa. *H3.3B* mutants were growth deficient and died at birth. *H3.3B* heterozygotes were also growth deficient, and the males were sterile as a result of developmental arrest of round spermatids (Tang *et al*. 2015). In another report, a *H3.3B* knockout (KO) mouse model resulted in a reduction in H3.3 histone levels leading to male infertility, in addition to abnormal sperm and testes morphology (Yuen *et al*. 2014). There was increased apoptosis in *H3.3B*-null germ cell populations at specific stages of spermatogenesis, and *H3.3B*-null testes displayed abnormal germ cell chromatin reorganisation and reduced protamine incorporation. Furthermore, disruption of *H3.3B* altered histone post-translational modifications and gene expression in the testes, with the most noticeable changes occurring in genes associated with spermatogenesis, demonstrating an important role for H3.3 in spermatogenesis (Yuen *et al*. 2014).

### Preparation of parental genomes – oogenesis

Oogenesis begins *in utero*, and the oocyte undergoes two asymmetric meiotic divisions during its maturation. In the prenatal period, oocytes only complete the first part of the first meiotic division. After a stint of active transcription during growth, oocytes are arrested in the prophase meiosis I as transcriptionally inactive germinal vesicle (GV) oocytes until the onset of puberty. At this stage, the nucleus is visible and contains a distinctive nucleolus (Swain & Pool 2008). This unusual phenomenon of the female germ line may be a protective mechanism against oxidative stress and DNA damage (Mira 1998). In response to a preovulatory surge of gonadotropin, the oocyte resumes meiosis, which is reliant on maternally synthesised RNAs and proteins. During meiotic nuclear maturation, as the GV oocyte exits from prophase (meiosis I) arrest, the nuclear envelope (NE) breaks down (also known as GV breakdown, GVBD), chromosome recombination and condensation takes place, and microtubule organising centres form a bipolar spindle to allow homologues to attach to the spindles at their centromeres (Eppig 1996, Swain & Pool 2008). Separation and segregation of homologues take place as they are pulled towards opposite poles by the meiotic spindle, resulting in unequal cytokinesis and extrusion of half their genetic material within the first polar body. Meiotic maturation progresses with spindle reassembly, until it stops and arrests at metaphase of meiosis II (MII), and is now referred to as a secondary oocyte (Swain & Pool 2008). The MII egg is now ready for fertilisation.

During oogenesis, there is widespread transcriptional changes and *de novo* DNA methylation, allowing the oocyte to obtain fertilisation and embryogenesis competency (Tomizawa *et al*. 2012). A number of post-translational histone modifications or histone remodelling help direct *de novo* methylation events in the oocyte, independent of DNA methylation maintenance, between cell divisions (Fig. 1a). DNA methylation in oocytes predominantly occurs in gene bodies, and it has been recently demonstrated that transcription events dictate DNA methylation sites and timing. However, it has been suggested that DNA methylation in the oocyte may only be necessary for imprinted genes (Stewart *et al*. 2015). Histone H3 lysine 4 (H3K4) trimethylation (H3K4me3) and dimethylation (H3K4me2) typically delineate sites of transcription initiation and are also hallmarks of CpG-dense regions known as CpG islands (CGIs) (Illingworth *et al*. 2008, Deaton & Bird 2011, Henikoff & Shilatifard 2011). Another histone mark, H3K36 trimethylation (K36me3), is associated with elongating eukaryotic chromatin (Edmunds *et al*. 2008).

A recent study investigating the histone modifications that may be implicated in promoting or inhibiting DNA methylation in oocytes, showed that CGIs destined for DNA methylation had reduced protective H3K4me2 and H3K4me3 in both primary and growing oocytes, whereas H3K36me3 increased specifically at these CGIs in growing oocytes (Stewart *et al*. 2015) Furthermore, methylome profiling of oocytes deficient in H3K4 demethylase KDM1A or KDM1B demonstrated that the removal of H3K4 methylation is required for proper methylation establishment at CGIs and that stepwise modulation of CGI chromatin facilitates DNA methylation acquisition (Stewart *et al*. 2015). In addition, continuous histone replacement and chromatin homeostasis play critical roles in transcriptional regulation and normal developmental progression. The replacement histone variant H3.3 (which replaces H3 and is incorporated into chromatin independent of DNA synthesis) has been identified as an essential maternal factor for oocyte reprogramming (Lin *et al*. 2014, Nashun *et al*. 2015) (Fig. 1b). In another recent report by Nashun and coworkers, a mouse oocyte-specific KO of the H3.3 chaperone *Hira* was developed to investigate histone turnover during oogenesis. Depletion of *Hira* in primordial oocytes caused a severe developmental defect and extensive oocyte death due to lack of continuous H3.3/H4 deposition, leading to abnormal chromosomal structure. These defects led to a decrease in the dynamic range of gene expression, the presence of invalid transcripts and unsuccessful *de novo* DNA methylation (Nashun *et al*. 2015), highlighting the importance of H3.3 in oocyte reprogramming.

### Small RNA species

Key regulatory molecules of small RNA biogenesis have been studied throughout spermatogenesis and oogenesis in mice (Luo *et al*. 2016). Dicer is responsible for the generation of both miRNAs and endo-siRNAs, which can post-transcriptionally silence gene expression in association with the ARGONAUTE (AGO) family of proteins (Stein *et al*. 2015). Conditional deletion of the RNase III enzyme, Dicer, can result in both male and female infertility (Murchison *et al*. 2007, Korhonen et al. 2011, Wang *et al*. 2015). In male mice, deletion of Dicer causes the disruption of spermatogenesis as a result of spermatocyte and spermatid depletion, leading to oligoteratozoospermic or azoospermic phenotypes (Wu *et al*. 2012). Similar phenotypes have been observed upon deletion of Drosha, another RNase III enzyme responsible for miRNA production (Wu *et al*. 2012).

In female mice, conditional knockout of Dicer causes meiosis I defects (Murchison *et al*. 2007). In contrast to the critical roles of miRNAs in male germ cells, surprisingly, the role of miRNAs during oogenesis is dispensable. Oocyte-specific deletion of Drosha (Yuan *et al*. 2016) or Dgcr8 (a cofactor of Drosha) (Suh *et al*. 2010) demonstrated no discernible phenotypic change. Taken together, the effects of Dicer deletion (miRNAs and endo-RNAs) and Dgcr8/Drosha deletion (miRNAs) indicate that endo-siRNAs might be the critical small RNA class in female meiosis. This has been demonstrated in a recent report by Paula Stein and coworkers, whereby disrupting siRNA function impairs meiotic maturation, spindle formation and chromosome alignment, leading to meiotic failure, and thereby highlighting that endo-siRNAs are indispensable during meiosis I in female mice (Stein *et al*. 2015).

The third class of small RNAs, the piRNAs, also plays key roles in spermatogenesis. The piRNA-associated proteins Mili (miwi-like) and Miwi2 (mouse piwi 2) are essential for spermatogonial stem cell formation and meiosis I progression, and Miwi is implicated in spermatid formation. Moreover, Miwi knockout mice exhibit male sterility, further emphasising the crucial role of piRNAs in spermatogenesis (Carmell *et al*. 2007, Thomson & Lin 2009). Furthermore, silencing of transposable elements occurs during male gametogenesis, via *de novo* DNA methylation of their regulatory regions, and loss of Mili and Miwi2 causes reduced piRNA expression and may thus be important in the establishment of *de novo* DNA methylation of retrotransposons in male germ cells (Kuramochi-Miyagawa *et al*. 2008). However, there are no known defects for piRNA-related protein mutants in female gametogenesis (Thomson & Lin 2009).

## Get set…

### Fertilisation and chromatin reprogramming

Mammalian fertilisation has six distinct stages, which include cumulus penetration, sperm-oocyte binding, fusion, oocyte inactivation, sperm processing and pronucleus (PN) formation (Swain & Pool 2008). Firstly, sperm must penetrate the cumulus mass and zona pellucida and enter into the oocyte cytoplasm. For the sperm-oocyte fusion to take place, the oocyte is activated by a sperm-oocyte-activating factor, such that it can undergo its second meiotic division (partly dependent on intracellular calcium [Ca^2+^] oscillations) and release a polar body. The parental genomes remain separated in the zygote. This is followed by extensive chromatin reprogramming, whereby the highly compacted protamine-associated sperm chromatin is removed by oocyte factors and equipped with new histones (Fig. 1b). In response to Ca^2+^ oscillations, a cortical reaction is induced (cortical granules migrate towards the oolemma to release enzymes into the perivitelline space), thereby preventing additional sperm binding and polyspermia (Ducibella *et al*. 1990, Schroeder *et al*. 1990). Spermatozoa must also undergo biochemical remodelling that is reliant on endogenous resources located within the oocyte cytoplasm, whereby decondensation of the sperm head takes place, releasing the sperm nuclear contents into the oocyte cytoplasm, in preparation for PN formation (Swain & Pool 2008). Moreover, the sperm membrane protein IZUMO1 and its oocyte receptor JUNO have been recently identified as critical factors for mammalian sperm-oocyte interaction, fusion, fertilisation and polyspermy prevention (Bianchi & Wright 2014, Bianchi *et al*. 2014, Grayson 2015). Finally, the formation of the maternal and paternal pronuclei delineates the completion of mammalian fertilisation (Swain & Pool 2008).

### PN formation

Mammalian sperm and oocyte epigenomes are characterised by gamete-specific 5mC patterns, which are reprogrammed during early embryogenesis. The parental genomes remain separated in the zygote (Fig. 1b). PN formation involves the re-establishment of the NE around the corresponding genetic material from the sperm and oocyte (Swain & Pool 2008). Firstly, there is a fusion of membrane vesicle, with successive incorporation of the nuclear pore complexes into the emergent NE, followed by transportation of lamins to create the underlying nuclear lamina scaffold (Macaulay & Forbes 1996, Swain & Pool 2008). The male PN forms centrally within the human oocyte, and the female PN forms adjacent to the second polar body (Grayson 2015). Subsequently, the female PN moves towards the central location of the male PN, which increases in size as a result of oocyte-derived vesicle membrane aggregation and fusion, as well as the addition of lamin B (Swain & Pool 2008). The NE is made up of a network of nuclear lamins that are regulated by chromatin interactions and covered by an inner and outer membrane. NE integrity is regulated through phosphorylation and dephosphorylation of nuclear lamins (Macaulay & Forbes 1996, Swain & Pool 2008). NE integrity and PN formation are determined by the activity of a number of oocyte-derived protein kinases and phosphatases, accumulated during oocyte maturation (Stuurman *et al*. 1998, Swain & Pool 2008).

The PN formation process has been classified into 6 stages, PN_0_−PN_5_ (Fig. 1b and c). In this section, we will describe the early PN stages: **PN_0_** = sperm entry immediately followed by completion of meiosis, formation of respective haploid pronuclei, sperm decondensation and elimination of the second polar body; **PN_1_** = PN sizes are comparable and there is an initiation of demethylation; **PN_2_** = active demethylation takes place in the paternal genome, whereas the maternal genome remains resistant (Swain & Pool 2008). The late PN stages will be discussed in the final section.

### Protamine-to-histone exchange

When a sperm enters an egg, the protamines are removed mainly by unknown maternal factors, and maternal histones are incorporated into the sperm DNA to establish *de novo* nucleosomes (Nonchev & Tsanev 1990, van der Heijden *et al*. 2008) (Fig. 1b). Although protamine removal and the subsequent sperm DNA decondensation are likely to be independent of histone deposition, *de novo* nucleosome assembly is Hira/H3.3 dependent and is essential for NE formation and the assembly of nuclear pore complexes (NPCs) during paternal PN formation (Inoue & Zhang 2014, Lin *et al*. 2014). The formation of the central H3.3/H4 is the first step for establishing *de novo* nucleosomes during preimplantation development (Inoue & Zhang 2014, Lin *et al*. 2014, Nashun *et al*. 2015). Histone H2A and H2B variants have also been implicated in genomic remodelling and sperm decondensation after fertilisation (Zalensky *et al.* 2002, Nashun *et al*. 2010, Tessarz *et al*. 2014), although exact mechanisms have not yet been defined. The incorporation of H2A/H2B variants may be replication dependent or could be encoded by alternative splicing of mRNA (Rai *et al*. 2014).

In mice, Hira-mutant zygotes present with a single PN (1PN) phenotype that is reminiscent of a phenomenon associated with human *in vitro* fertilisation (IVF) and intracytoplasmic sperm injection (ICSI). It has been indicated that 2.7−17% of all ICSI/IVF procedures produce 1PN zygotes, with one-third of these 1PN phenotypes arising as a result of paternal PN formation failure (Azevedo *et al*. 2014). The underlying mechanisms for the failure of these zygotes to progress into the 2PN stage are not well understood. A recent study has observed that 1PN zygotes have the least developmental potential to form blastocysts compared with other clinically discarded human embryos (Yao *et al*. 2015). It will be of interest to collect human abnormal 1PN zygotes and investigate whether Hira-mediated H3.3 incorporation is conserved for PN formation across species.

### The role of small RNAs during fertilisation

During fertilisation, sperm deliver a series of small RNAs into the oocyte (Krawetz 2005), and the roles of these sperm-derived miRNAs and endo-siRNAs have only recently been uncovered (Yuan *et al*. 2016). In this study by Yuan and coworkers, although sperm retrieved from Dicer- and Drosha-mutant mice (with altered miRNA and endo-siRNA profiles) could fertilise wild-type oocytes when introduced via intracytoplasmic sperm injection (ICSI), there was an evidence of significant reduction in the developmental potential in the zygote, from 2PN formation to the two-cell embryo stage. However, embryonic development could be rescued by introducing wild-type total or small RNAs into ICSI embryos, demonstrating the specific requirement of paternal small RNAs during fertilisation and preimplantation embryonic development (Yuan *et al*. 2016).

## Go!

### Late PN mitosis and first cleavage

The late PN stages can be described as follows: **PN_3_** = demethylation is complete in the paternal genome, **PN_4_** = the two pronuclei move closer together and **PN_5_** = maternal and paternal pronuclei are adjacent to each other before syngamy (Swain & Pool 2008) (Fig. 1c). After fertilisation, there is a higher transcriptional activity in paternal PN with a greater concentration of transcription factors, due to a more transcriptionally permissive chromatin structure than that in the maternal PN (Worrad *et al*. 1994, Aoki *et al*. 1997, Schultz 2002, Johnson *et al*. 2011). Genome-wide chromatin reprogramming of the paternal genome is predominantly controlled mainly by unknown maternal factors (Gu *et al*. 2011). However, we have recently demonstrated that maternal Hira, and in turn H3.3 incorporation, is compulsory for mouse development past the zygote stage and that Hira/H3.3-dependent transcription of ribosomal RNA (rRNA) is essential for first cleavage. Furthermore, our study also showed great reduction in DNA replication and transcription in parental genomes of Hira mutants, corroborating that transcription is needed for zygote development (Lin *et al*. 2014). Although the post-fertilisation replacement of protamines with histones is not well understood, it is evident that sperm chromatin decondensation is a prerequisite (Jenkins & Carrell 2012).

### DNA demethylation and epigenetic asymmetry

During embryonic development, DNA methylation provides an epigenetic regulatory mechanism for the differentiation of cells towards their future lineages, while preventing their regression into an undifferentiated state (Messerschmidt *et al*. 2014). Conversely, DNA demethylation is also essential in the preimplantation embryo to permit sexual reproduction and in establishing pluripotency, and a second wave of global epigenetic reprogramming takes place with methylation levels being at their lowest by the early blastocyst stage (Messerschmidt *et al*. 2014).

DNA replication in the zygote has been demonstrated as one of the key processes for demethylation of DNA (Fig. 1b). In line with our findings which showed that maternal Hira/H3.3 is upstream of DNA demethylation and is essential for DNA replication (Lin *et al*. 2014), recent studies have demonstrated the reduction of both DNA replication (Nashun *et al*. 2015, Tang *et al*. 2015) and DNA demethylation in pronuclei (Nashun *et al*. 2010) in double H3.3 KO mice and in a Hira-mutant line. A newly developed measurement approach using mass spectrometry showed that a *de novo* DNA demethylation event occurs during the early pronuclear stage before DNA replication and is therefore independent of DNA replication (Amouroux *et al*. 2016). Further investigation is necessary to obtain a comprehensive understanding of the relationship between histone marks and the dynamics of DNA demethylation.

Studies in mice have shown that active demethylation of the male PN is completed within 4 h of fertilisation, immediately after sperm decondensation, and is not independent of PN formation (Santos *et al*. 2002). After sperm decondensation, the paternal genome predominantly displays active DNA demethylation, with progressive accumulation of 5hmC marks and is independent of DNA replication. However, recent evidence suggests that in addition to active mechanisms, passive activities may also contribute to the demethylation of the paternal genome. In passive demethylation, the nascent DNA strand remains unmethylated after replication, and methylation is lost over time through subsequent DNA replication and cell division. At this stage, maternal and paternal genomes are unequally methylated. The maternal genome remains relatively stable at this point in time (preserving histone modifications acquired from the time of oocyte growth), with the exception of marks associated with transcription and/or replication (Burton & Torres-Padilla 2010). Active demethylation of the maternal genome is protected from Tet3 oxidation by developmental pluripotency-associated protein 3 (Dppa3, also known as PGC7) (Nakamura *et al*. 2007). However, it has been recently reported that extensive active and passive demethylation takes place in both parental genomes before the first mitotic division and is likely to be mediated by demethylation mechanisms downstream of Tet3 oxidation (Guo *et al*. 2014). There is subsequent *de novo* methylation after implantation, which may be important for early lineage specification (Santos *et al*. 2002).

Epigenetic asymmetry in the preimplantation embryo may be associated with differences in transcriptional timing and the regulation of chromatin architecture in the parental pronuclei (Burton & Torres-Padilla 2010). Asymmetric epi-marks at several imprinted gene loci are maintained to allow parent-of-origin-specific gene expression in the embryonic tissue (Feil 2009, Nakamura *et al*. 2007). Moreover, attainment of the hyperacetylated and hypermethylated chromatin state of the paternal genome may allow easy access and remodelling during early embryogenesis.

### Zygotic genome activation

Following fertilisation, maternal-to-zygotic transition takes place, whereby oocyte-derived mRNAs are degraded and transcription of the maternal and paternal genomes, or zygotic genome activation (ZGA), is initiated. ZGA plays an essential role in preimplantation development, and it is widely accepted that there are two waves of ZGA: major ZGA and minor ZGA. Recent reviews have extensively evaluated and provided insights into ZGA (Lee *et al*. 2014, Abe *et al*. 2015, Ko 2016). Minor ZGA occurs during the late pronuclear stage and is followed by the major ZGA wave during the 2-cell embryonic stage in mice and the 4–8-cell stages in humans (Ko 2016). It has been suggested that minor ZGA is not required for embryonic development. Recent evidence has demonstrated that although oocyte nucleolar precursor bodies (NBPs; oocyte nucleoli, where rRNA production takes place) are essential for embryonic development, zygotic NPBs may not be (Kyogoku *et al*. 2014), as rRNA production and processing are not controlled by zygotic NPBs (Fulka & Langerova 2014, Fulka & Aoki 2016). However, we have recently demonstrated that minor ZGA, and particularly RNA polymerase I transcription during the early pronuclear stage, is critical for embryo cleavage (Lin *et al*. 2014). By contrast, a study by Kone and coworkers reported that embryos subjected to pharmacological inhibition of RNA polymerase I during the late pronuclear stage (when there is maximal DNA synthesis) reached the blastocyst stage (Kone *et al*. 2016). This discrepancy could be due to the timing of treatment and may also suggest that polymerase I transcription in the zygote is initiated before the late PN stages. Moreover, the minor ZGA wave has recently been suggested as an active component of chromatin remodelling in 1-cell embryos (Abe *et al*. 2015). It has also been demonstrated that nucleolar structure relies on rRNA accumulation (Falahati *et al*. 2016), and small and dispersed NPBs with reduced DNA replication have been reported in H3.3 double knockout zygotes (Tang *et al*. 2015), further highlighting the importance of rRNA in zygotic cleavage. Furthermore, mechanical removal of NPBs in GV stage oocytes in a recent study did not affect the rRNA levels, indicating either (a) that rDNA is probably no longer located inside NPBs or (b) the existence of unidentified novel rRNA transcription machineries in zygotes (Fulka & Langerova 2014, Fulka & Aoki 2016). Further detailed analyses and screening is required for the identification of these potential regulators. A new and unbiased approach, using end-targeting proteomics of isolated chromatin segments (ePICh) of a mammalian rRNA gene promoter, has identified new factors bound to the promoter region of the rRNA genes in mouse erythrocyte leukaemia cells (Ide & Dejardin 2015). By adopting and optimising this ePICH technology, it would be useful to characterise any molecules bound to the promoter region of the rRNA genes in the oocyte.

As described previously, there is a retention of histones in sperm that exhibit a significant enrichment upstream of rRNA sequences in mice (Johnson *et al*. 2016), and inherited paternal chromatin may provide a preferentially accessible structure in the paternal PN that is necessary for rRNA transcription and utilisation by the preimplantation embryo (Steger 1999, De Vries *et al*. 2012, Rathke *et al*. 2014). Interestingly, the enhancer-bound regulatory protein Ctcf, which may permit fast remodelling of chromatin organisation in preparation for ZGA, is not present in human sperm, suggesting that post-fertilisation transcriptional regulation is species specific (Johnson *et al*. 2016). It will be interesting to further study rRNA transcription in the human zygote, and abnormal 1PN zygotes could serve as a model.

Oocytes are enriched in endo-siRNAs and piRNAs, and there are dramatic changes in the expression profiles of different RNA species during oocyte-to-egg transition, and after ZGA, the embryo is enriched with miRNAs (Ohnishi *et al*. 2010). Furthermore, beyond the three major species of small RNAs, the role of transfer RNAs (tRNAs) in the regulation of retroviral elements in preimplantation embryos has recently been identified (Sharma *et al*. 2016). However, despite numerous investigations into individual small RNA species, the functions and dynamics of stage- and cell-specific RNA clusters remain largely unknown. Reporter systems can serve as an excellent model to monitor spatial–temporal dynamics and functional mechanisms (Parchem *et al*. 2014), and it will also be worthwhile to investigate the non-canonical functions of nuclear Dicer and Drosha during epigenetic reprogramming of the zygote (Burger & Gullerova 2015).

## Conclusion and perspectives

Epigenetic reprogramming in the zygote is a highly dynamic process, with tightly coordinated cellular and molecular events occurring within a few hours of fertilisation. The development of single-cell epigenomic approaches, such as the combination of chromatin immunoprecipitation and next-generation sequencing (including RNA-seq and ChIP-seq) and genome-wide bisulfite sequencing (Smallwood *et al*. 2011, Rotem *et al*. 2015, Angermueller *et al*. 2016, Clark *et al*. 2016), are powerful tools that will accelerate our understanding of the zygotic epigenetic reprogramming that takes place during the stages described previously. Moreover, the application of newly developed techniques to investigate both preceding and subsequent stages, including gametogenesis − with particular focus on oocyte maternal factors (Li *et al*. 2010, Condic 2016), as well as preimplantation, peri-implantation and postimplantation embryos (Deglincerti *et al*. 2016, Shahbazi *et al*. 2016), will be valuable for the development of potential therapeutic targets for infertility and for improving clinical outcomes.

## Declaration of interest

The authors declare that there is no conflict of interest that could be perceived as prejudicing the impartiality of the research reported.

## Funding

This work was supported by the Medical Research Council (grant number: G1002033) and the Wellcome Trust’s Institutional Strategic Support Fund (ISSF) at the University of Edinburgh.
